# Evolutionary Conservation of Thyroid Hormone Receptor and Deiodinase Expression Dynamics *in ovo* in a Direct-Developing Frog, *Eleutherodactylus coqui*

**DOI:** 10.3389/fendo.2019.00307

**Published:** 2019-05-24

**Authors:** Mara Laslo, Robert J. Denver, James Hanken

**Affiliations:** ^1^Department of Organismic and Evolutionary Biology, and Museum of Comparative Zoology, Harvard University, Cambridge, MA, United States; ^2^Departments of Molecular, Cellular and Developmental Biology, and Ecology and Evolutionary Biology, University of Michigan, Ann Arbor, MI, United States

**Keywords:** embryo, direct development, thyroid hormone, amphibians, evolution, metamorphosis, maternal effects, life history

## Abstract

Direct development is a reproductive mode in amphibians that has evolved independently from the ancestral biphasic life history in at least a dozen anuran lineages. Most direct-developing frogs, including the Puerto Rican coquí, *Eleutherodactylus coqui*, lack a free-living aquatic larva and instead hatch from terrestrial eggs as miniature adults. Their embryonic development includes the transient formation of many larval-specific features and the formation of adult-specific features that typically form postembryonically—during metamorphosis—in indirect-developing frogs. We found that pre-hatching developmental patterns of thyroid hormone receptors alpha (*thra*) and beta (*thrb*) and deiodinases type II (*dio2*) and type III (*dio3*) mRNAs in *E. coqui* limb and tail are conserved relative to those seen during metamorphosis in indirect-developing frogs. Additionally, *thra, thrb*, and *dio2* mRNAs are expressed in the limb before formation of the embryonic thyroid gland. Liquid-chromatography mass-spectrometry revealed that maternally derived thyroid hormone is present throughout early embryogenesis, including stages of digit formation that occur prior to the increase in embryonically produced thyroid hormone. *Eleutherodactylus coqui* embryos take up much less 3,5,3′-triiodothyronine (T_3_) from the environment compared with *X. tropicalis* tadpoles. However, *E. coqui* tissue explants mount robust and direct gene expression responses to exogenous T_3_ similar to those seen in metamorphosing species. The presence of key components of the thyroid axis in the limb and the ability of limb tissue to respond to T_3_ suggest that thyroid hormone-mediated limb development may begin prior to thyroid gland formation. Thyroid hormone-dependent limb development and tail resorption characteristic of metamorphosis in indirect-developing anurans are evolutionarily conserved, but they occur instead *in ovo* in *E. coqui*.

## Introduction

**Figure 1:**
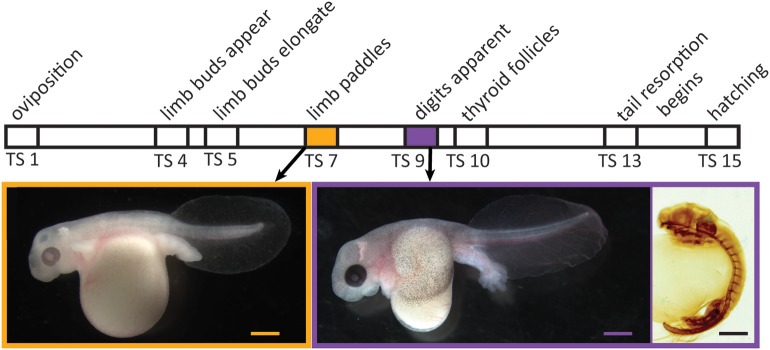
Relative timing of several developmental events during embryogenesis in *Eleutherodactylus coqui*. Images depict live TS stage 7 **(Left)** and stage 9 **(Middle)** embryos removed from overlying egg membranes, and a TS stage 9 embryo **(Right)** stained for type II collagen, which reveals the cartilaginous skeleton and notochord. Scale bars, 1 mm.

**Figure 2:**
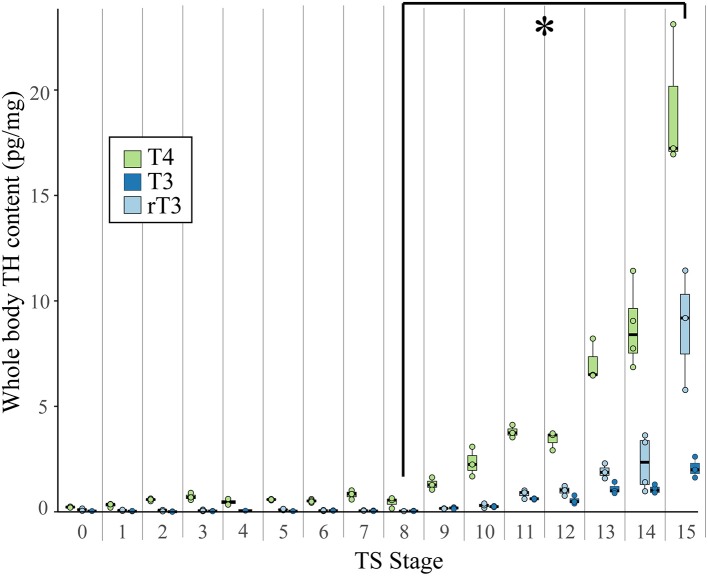
Whole body content of T_4_, T_3_, and rT_3_ in pooled *E. coqui* embryos at TS stages 1–15 and in unfertilized oocytes (TS 0) as quantified by LC-MS/MS. Whole body content of iodothyronines was normalized to sample weight; between 15 and 20 embryos were pooled to generate one biological replicate. Each value depicted in the graph is based on two-to-four replicates. Values based on fewer than three replicates are not included in the statistical analysis. All three iodothyronines increased significantly between TS stages 8 and 15 (*post-hoc* Dunn's test; *p* < 0.05), indicated by the asterisk (*). See Supplementary Data for a complete list of significant pairwise differences. Each boxplot represents median and range of the data.

**Figure 3:**
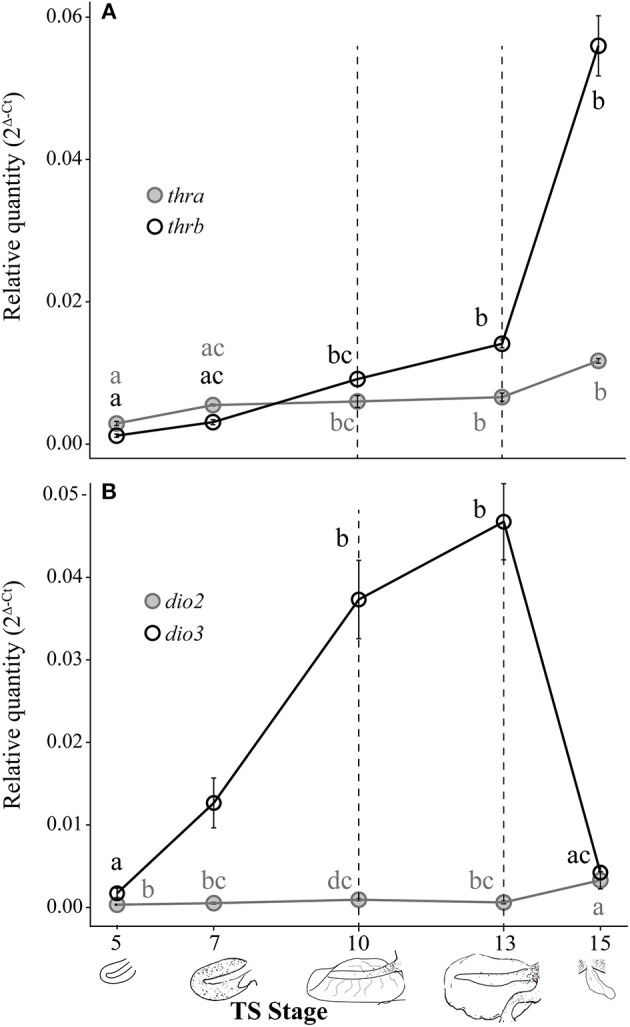
Relative *thra* and *thrb* mRNA levels **(A)** and *dio2* and *dio3* mRNA levels **(B)** in the pre-hatching tail of *E. coqui*. Dashed vertical line at TS stage 10 marks when thyroid follicles are first visible in histological sections; the line at TS 13 indicates the onset of tail resorption. Drawings on the x-axis depict tail growth and resorption before hatching. Each expression value is represented as a circle centered on the mean of 3–7 individuals ± SE. Lower-case letters in gray (*thra* and *dio2*) and black (*thrb* and *dio3*) indicate significant pairwise differences between groups (*post-hoc* Dunn's test, *p* < 0.05). See Supplementary Data for a complete list of pairwise comparisons.

Direct development, a distinctive life-history mode in amphibians and other animals, has evolved in anurans multiple times from the ancestral biphasic life history; it characterizes many hundreds of living species (1). Even though direct-developing frogs typically lack both a free-living aquatic larval stage and a discrete, post-hatching metamorphosis, many species display a cryptic metamorphosis before hatching: adult-specific features, such as limbs, form precociously in the egg, and numerous tadpole-specific features are present initially but then are lost [Figure 1; (2, 3)]. Because such changes in frogs with indirect development are mediated by thyroid hormone (TH), the primary regulator of metamorphosis (4), evolutionary change in thyroid axis function and timing may underlie the numerous heterochronies observed between direct-developing and indirect-developing species (5–9). Yet, there have been few attempts to precisely delineate the role of this or other pertinent physiological mechanisms.

Embryonic development of direct-developing frogs, as seen in the Puerto Rican coquí, *Eleutherodactylus coqui*, appears to comprise a mosaic of TH-independent and TH-dependent features. We use the term “embryonic” to describe all *in ovo* development in *E. coqui*, although this period encompasses both the initial formation of major organ systems as well as the patterning, morphogenesis and growth that follows. Many of the latter events correspond to metamorphic changes in biphasic anurans. It was once thought that embryonic development in direct-developing species was primarily TH-independent (5). However, subsequent studies with exogenous T_3_ and with TH-synthesis inhibitors suggested at least a partial role for TH in terminal stages of limb development as well as tail resorption (6, 10). In *E. coqui*, for example, treatment with exogenous T_3_ causes precocious tail resorption but has little to no effect on limb elongation (11). Similarly, treatment with methimazole, a TH-synthesis inhibitor, inhibits only tail resorption and late stages of limb elongation but does not affect early limb differentiation or digit formation (8). The apparent TH-independence of early stages of limb development is correlated with the fact that limb bud, paddle and digit formation occur prior to formation of the embryonic thyroid gland [Figure 1; (12, 13)]. Thus, limb development in *E*. *coqui* comprises two periods: limb bud differentiation and paddle and digit morphogenesis, which precede formation of the thyroid gland and may be TH independent; and limb growth and elongation, which follow thyroid gland formation and are TH dependent. Experiments with TH-synthesis inhibitors, however, can only address the role of TH in the second period. The presumed TH independence of the first period remains to be verified experimentally.

All organs in the body are exposed to roughly the same concentration of circulating TH, primarily in the form of thyroxine (T_4_) and lower concentrations of 3,5,3′-triiodothyronine [T_3_; (14, 15)]. Hereafter, we use the term TH to refer to both T_4_ and T_3_. However, tissue-specific differences in uptake, metabolism, and action provide for diverse effects of TH in different tissues. Thus, tissue-specific changes in TH metabolism and action likely contribute to the heterochrony of developmental events observed in direct-developing anurans relative to biphasic species. Alternatively, the principal locus of change in hormonal control may involve a shift in the source of THs and when they are present in the embryo. Maternally derived TH is present at early developmental stages of all vertebrates examined so far. In most vertebrates, maternal TH is in the yolk; in most mammals, maternal TH can pass from mother to fetus via the placenta or milk. Yet, the role of maternally derived TH in amphibian embryos is poorly understood (16–18). If maternally derived THs are present in early embryos of *E. coqui*, they could influence limb development prior to formation of the embryonic thyroid gland. Finally, three different deiodinase enzymes control cellular metabolism of T_4_ in target tissues. In amphibians, two types of deiodinases play major roles during development. Deiodinase enzyme type II (Dio2) converts T_4_ into T_3_, which has at least 10 times greater affinity for TH receptors (TRs) than T_4_. Deiodinase type III (dio3) converts T_4_ to both T_2_ and reverse triiodothyronine (rT_3_), which are unable to bind TRs in most species. Thyroid hormones act by binding to two TR subtypes, designated alpha (α) and beta (β), to activate or repress transcription of TH target genes. Contrasting expression patterns of TRs and deiodinases may in part underlie the diverse, tissue-specific effects of TH in *Xenopus* species (19–26), and it is likely that changes in the temporal or spatial expression of deiodinases or TRs influence TH competence and action in target tissues in *E. coqui*.

Here we tested the hypothesis that developmental changes in TR and deiodinase mRNAs in developing *E. coqui* limb and tail, and in whole body TH content are conserved relative to those seen during metamorphosis in indirect-developing frogs. We also investigated whether *E. coqui* tissues are capable of responding directly to T_3_ action by mounting gene regulation responses similar to those seen in metamorphosing species. Taken together, our data support the hypothesis that limb development and tail resorption in *E. coqui* (8, 12) are mediated by conserved components of TH signaling. Additionally, our results suggest that maternal TH could facilitate limb development prior to formation of the embryonic thyroid gland.

## Materials and Methods

### Animal Care

Live adult *Eleutherodactylus coqui* were field-collected from introduced populations in Hilo, Hawaii, with the permission of the U.S. Fish and Wildlife Service (permits EX-14-06, EX-16-07, and EX-17-11). They were brought to Harvard University and maintained as a breeding colony in the Hanken laboratory (IACUC protocol #99-09-03); embryos were obtained following spontaneous matings. Following removal of the overlying chorion with watchmaker forceps in 2% cysteine (pH 8.5) in 10% Holtfreter solution, embryos were reared in 10% Holtfreter solution in Petri dishes at 22.5°C. Embryos were staged according to the normal table of Townsend & Stewart (TS; 1985), which defines 15 stages from fertilization (1) to hatching (15). Following internal fertilization, the adult female deposits embryos at TS stage 1.

### Molecular Cloning and Sequence Validation

Partial cDNAs for *dio2, dio3, thra, thrb, ribosomal protein L8* (*rpL8*), *thyroid hormone induced bZip protein* (*thibz*), and *alpha-actinin 4* (*actn4*) (Genbank accession numbers MK784754, MK784753, MK784748, MK784749, MK784751, MK784750, MK784755) were isolated by PCR with exact primers (Table 1) using cDNA generated from RNA isolated from whole TS stage 13 embryos, and the resultant DNA fragments were subcloned into the pCR II plasmid. Exact primers for *dio2, dio3, thra, thrb, rpL8*, and *thibz* were designed from predicted full-length cDNA sequences provided by L. Sachs, N. Buisine, and G. Kerdivel (personal communication), while *actn4* primers were designed from genomic sequences provided by A. Mudd, R. Harland, and D. Roksahr (personal communication). We also subcloned a partial cDNA for *krüppel-like factor 9* (*klf9*) by degenerate PCR (oligonucleotide primers designed using CODEhop) using the same cDNA described above (Genbank accession number MK784752). The sequences of the subcloned partial cDNA fragments were confirmed by direct DNA sequencing and by comparing them against the full-length cDNAs provided by the investigators listed above.

**Table 1:** Degenerate PCR, exact PCR, and qPCR primers and probes for *Eleutherodactylus coqui* and *Xenopus tropicalis*.

**Gene**	**Type**	**Species**		**Sequence**	**Probe sequence**	**Amplicon size (bp)**
*thibz*	qPCR	*E. coqui*	F	GAGGGTCAAACGCCAGTATT	TGAAGGGTGCTATAAAGTAGCTGAT	72
			R	GTCCGGGTCTGTGTAATGTC		
*klf9*	qPCR	*E. coqui*	F	CAAGTCCTCCCACCTCAAAG	CCCACTACAGAGTGCATACAGGTGA	65
			R	CATGTGCATGGAAATGGACG		
*rpL8*	qPCR	*E. coqui*	F	CTGGAGGTGGACGTATTGAC	ACCCATTCTGAAGGCAGGTCGT	68
			R	TCTTGGCCTTGTACTTGTGG		
*dio2*	qPCR	*E. coqui*	F	ACACAGTTACCTCAACAGGG	TGCAATCTGATCTCCCAGGAGCA	87
			R	AACAGTGTGGAACATGCAGA		
*dio3*	qPCR	*E. coqui*	F	GCAGCCCAGCAGTATTATCA	CGTGGAGGACATGCGTTTAACCC	95
			R	CACATGGGTGGTCTCGTTTA		
*thra*	qPCR	*E. coqui*	F	ACTACATCAACCACCGCAAA	CCCACTTCTGGCCTAAGCTCCT	81
			R	CAATCATGCGCAAGTCAGTC		
*thrb*	qPCR	*E. coqui*	F	GCAGCCCAGCAGTATTATCA	TCAAATGTTGTGCCTGCGGCT	95
			R	GTGATCACCATGGGAGATGG		
*actn4*	qPCR	*E. coqui*	F	AAGCCATCTCTGAAGTCCTC	AGTGCCAGCCTTCCTCAGGTG	80
			R	TTTCACGGCTTGGTGTAACT		
*rpL8*	qPCR	*E. coqui*	F	GACCAGAGTAAAGCTGCCTTCT	SYBR	95
			R	TTGTCAATACGTCCACCTCCAG		
*thra*	qPCR	*E. coqui*	F	CGACAAAATCACCCGAAATCAGT	SYBR	78
			R	GACAAGGTCCATTGCCATGC		
*thrb*	qPCR	*E. coqui*	F	CTTGCGCCTCTTTTCTCTGTTT	SYBR	76
			R	CAGATCTGGTTTTGGATGACAGC		
*klf9*	Degenerate	*E. coqui*	F	GGSTGTGGCAAAGTYTAYGGSAA		215
			R	TTGGTYAARTGRTCRCTCCTCAT		
*rpL8*	Exact	*E. coqui*	F	GACATTATCCATGATCCAGGCCG		616
			R	CAGTCTTTGTACCGCGCAGACG		
*dio2*	Exact	*E. coqui*	F	GAGTGTGGACCTGTTGATCACT		745
			R	TTTCTGTTCCATCCACTGTCGT		
*dio3*	Exact	*E. coqui*	F	TGCAAACTTCTCAAACAGGTGG		716
			R	TTCCTCAGTTCAGCGATCTTGT		
*thra*	Exact	*E. coqui*	F	AGAGCCAGATGAAAAGAGGTGG		801
			R	CTGTCAGGATCGTAACGCACA		
*thrb*	Exact	*E. coqui*	F	CTAGCAGCATGTCAGGGTACAT		779
			R	TACCACCCCTAGTCCTCCATTT		
*actn4*	Exact	*E. coqui*	F	GAAACAGCAGCGGAAGACTTTC		619
			R	CTTCTTATCAGGACGAGCGGTG		
*thibz*	Exact	*E. coqui*	F	CTCCATGATTCAACTCCACCCA		961
			R	CGTAGTGAGGGTGAGACAACAA		
*thibz*	qPCR	*X. tropicalis*	F	AAGAGACGCAAGAACAACGA	AGAAGCGCCGGGCGGGGGA	111
			R	GAGTCGGGCATTCTCTTCAA		
*klf9*	qPCR	*X. tropicalis*	F	AGTCTTCCCACCTTAAAGCC	ACGCCCTTTTCCGTGTACGTGGCCT	106
			R	GTCAACTCATCGGAACGAGA		
*eef1a1*	qPCR	*X. tropicalis*	F	CTTGACTGCATTTTGCCACC	AGCCTCTGCGTCTGCCTCTGCAGG	112
			R	GTCTCCACACGACCAACTG		
*dio3*	qPCR	*X. tropicalis*	F	CGGTGCCTACTTTGAGAGAC	TACCAGGGAGGGCGGGGGCC	94
			R	CCGAGATCTTGTAGCCTTCC		
*thrb*	qPCR	*X. tropicalis*	F	TTGATGATACCGAAGTCGCC	TCGCCCTGGCCTCACTAGTGTGGAGA	102
			R	AACCTTCCTGGCACTTTTCT		
*actn1*	qPCR	*X. tropicalis*	F	CAAAGTGCTGGCTGTCAATC	AGCTGGCCAGTGATCTGCTGGAGTGG	105
			R	TCTAACCAAGGGATTGTGCG		

Prior to the full-length predicted cDNA sequences becoming available, oligonucleotide primers for SYBR-based reverse transcriptase quantitative PCR (RTqPCR) were designed based on the available mRNA sequences on Genbank for *thra* and *thrb*, and the previously cloned *rpL8* [Genbank accession numbers AF201957.1 and AF201958.1; (8), Table 1]. For probe-based quantitative PCR (qPCR), primers and probes for *actn4* were designed from the partial cloned cDNA sequence while *dio2, dio3, thra, thrb, rpL8, thibz*, and *klf9* were designed based on the full-length sequences from other investigators listed above (Genbank accession numbers MK784763, MK784762, MK784757, MK784756, MK784760, MK784758, MK784759, MK784761).

### Whole Body Extraction and Quantification of Iodothyronines Using LC-MS/MS

The iodothyronines T_3_, rT_3_, T_4_, and T_2_ were quantified from whole *E. coqui* embryos throughout development. Because embryos were not dissected from the yolk, all measurements include embryo and yolk TH content. Animals at different stages were anesthetized and snap frozen until extraction and LC-MS/MS analysis. Unfertilized oocytes were dissected from the ovaries of a newly sacrificed female and snap frozen. Between 15 and 20 embryos (~ 600 mg) were pooled to make one biological replicate. Three or four biological replicates were used for each developmental stage. Tissues were extracted for thyroid hormone analysis as described by Denver (27, 28) with the following modifications: stable isotope-labeled T_3_ and T_4_ (^13^C_6_ T_3_ and T_4_, Sigma) were used as an internal standard to correct for differences in extraction efficiency, and solid phase extraction with a Supel-Select SCX cartridge (60 mg 3 mL, Sigma) was used to further purify the extracted tissue. After conditioning the cartridge with 3 mL methanol (HPLC Grade, Sigma) and equilibrating it with 5 mL of 2% formic acid in water (HPLC Grade, Sigma), the sample was loaded, rinsed first with 3 mL 2% formic acid in water and then with 3 mL methanol, and finally eluted with 2 mL of freshly prepared 5% ammonium hydroxide in methanol. It was then evaporated to dryness under nitrogen flow and resuspended in 100 μl of 0.1% formic acid in methanol. Samples were measured at the Harvard Small Molecule Mass Spectrometry facility by using gradient liquid-chromatography mass-spectrometry (LC-MS/MS). Ten microliters of samples were injected on a C18 column (Kinetex 2.6 μm, 100 Å pore size, 150 × 2.1 mm, Phenomenex) in an Agilent 1290 HPLC coupled with an Agilent 6460 Triple Quad Mass Spectrometer. See Supplementary Information for the LC and MS parameters (Supplementary Tables 1, 2). Calibration curves were made in 0.1% formic acid in methanol with pure standards and the same amount of internal standard as the samples. Quantification results with a signal-to-noise (S/N) ratio >10 were used for the statistical analysis. Results with a ratio between 3 and 10 (purple type; Supplementary Table 3) were included in the graph (Figure 2) but not used in the statistical analysis; those with a ratio below 3 were not used (red type; Supplementary Table 3). We normalized iodothyronine content to the weight of the tissue extracted.

### Quantitative PCR

Dechorionated embryos were anesthetized by immersion in 10% Holtfreter solution with drops of 2% neutral-buffered MS-222 added until the embryos no longer responded to toe pinches (between 30 and 60 s). Limbs and tails were dissected and homogenized in TriZol reagent (Invitrogen) and kept at −20°C until RNA isolation. Total RNA was isolated following the manufacturer's protocol within 3 weeks of homogenization. Because qPCR primers did not span exon-exon boundaries, genomic DNA was removed with an Ambion DNA-*free* kit (cat. #AM1906). Controls with no reverse-transcriptase verified that removal of genomic DNA was complete. Total RNA was quantified with a Qubit Fluorometer 3.0 and checked for purity on a Nanodrop spectrophotometer. For SYBR Green RTqPCR assays, 200 ng of total RNA was used for input for each reaction. For probe-based qPCR, 660 ng of total RNA for each sample was synthesized into cDNA with iScript Reverse Transcriptase Supermix for RT-qPCR (BioRad). Complementary DNA was kept at −20°C until the qPCR assay was performed. mRNA levels were analyzed with either Ssoadvanced Universal Probes Supermix (BioRad) or an iTaq Universal SYBR Green One-Step kit (BioRad) on a CFX384 machine. See Supplementary Data for qPCR cycling conditions. Optimal qPCR conditions were determined with temperature gradient and cDNA dilutions for dynamic range of input. Standard curves showed high efficiency of reaction (90–105%), and *R*^2^ was equal to or >0.98 for all primer sets. No template controls showed no amplification. All oligonucleotides are listed in Table 1. All SYBR and probe-based qPCR experiments were done in simplex. The relative mRNA levels were determined as described by Schmittgen and Livak (29). For the developmental expression studies, target-gene expression was normalized to the reference gene *rpL8*, which did not show significant variation across development [*rpL8* mRNA values are given in Supplementary Table 5; see also (8)]. In the *in vivo* and the tissue explant T_3_ response experiments, *E. coqui* target gene mRNA levels were normalized to the reference genes *rpL8* and *actn4*, which was unaffected by T_3_ treatment. Small, statistically insignificant changes in reference gene mRNAs could have led to a small underestimation of the effect of T_3_ in these experiments.

For *Xenopus tropicalis*, qPCR primers and probes for *thrb, klf9, thibz, dio3, elongation factor 1 alpha* (*eef1a1*) and *alpha-actinin 1* (*actn1*) were designed from publicly available sequences (Genbank accession numbers XM_012964865.2, NM_001113674.1, XM_018092557.1, NM_001113667.2, NM_001016692.2, and NM_001079198.1). For tissue explant experiments, *X. tropicalis* target gene expression was normalized to *eef1a1* and *actn1*.

### Treatment of *E. coqui in vivo*

*Eleutherodactylus coqui* embryos were dechorionated into 10% Holtfreter solution at least 24 h prior to immersion in T_3_. One mM stock T_3_ in DMSO or 0.01 N NaOH was diluted to make 50 nM T_3_ in 10% Holtfreter solution. We chose 50 nM T_3_ because it has been shown to induce tail resorption in *E. coqui* (8), and a 46-h timepoint to allow enough time for induction of T_3_ response genes. We chose TS stage 9 embryos because the last third of limb development is TH-dependent (8), but TS stage 9 is still prior to thyroid gland activation. T_3_ treatment solutions were refreshed every 8–12 h. After 46 h (*n* = 12–14 TS-9 embryos), dechorionated embryos were anesthetized as described above and limbs and tails were dissected, from which total RNA was extracted using TriZol reagent.

### Measurement of Environmental T_3_ Uptake in *X. tropicalis* and *E. coqui*

To determine if *E. coqui* embryos are capable of taking up TH from their surrounding environment, we immersed dechorionated TS stage 9 *E. coqui* embryos or NF 51–55 *X. tropicalis* tadpoles in 30 mL (*E. coqui*) or at least 500 mL (*X. tropicalis*) 10% Holtfreter solution with either 1 nM (*n* = 4–6 biological replicates/treatment) or 50 nM (*n* = 3–4 biological replicates/treatment) stable isotope-labeled T_3_. We chose TS stage 9 *E. coqui* embryos to match the *in vivo* T_3_ treatment experiments and selected *X. tropicalis* tadpoles with developing limbs with similar morphology to *E. coqui* TS stage 9. Approximately twenty *E. coqui* individuals (600 mg tissue) or two tadpoles were pooled were pooled to make one biological replicate. Tadpoles were either ordered from Xenopus1 (Ann Arbor, Michigan, U.S.A.) or derived from the Hanken lab colony. Stock 100 μg/mL stable isotope-labeled T_3_ was diluted to either 1 or 50 nM T_3_. After either 8 or 24 h in 1 nM labeled T_3_ solution or 46 h in 50 nM T_3_ solution, *X. tropicalis* tadpoles and *E. coqui* embryos (with yolk removed) were anesthetized with neutral-buffered 2% MS-222, rinsed three times in PBS and snap frozen until extraction. On average, *E. coqui* embryos were more densely packed in T_3_ solution (5.9 mg tissue per mL media) than *X. tropicalis* tadpoles (2.0 mg tissue per mL media); however, *E. coqui* embryos are routinely cultured in these conditions with no ill effects. Tissue was extracted as described above. Because we measured whole body content of stable isotope-labeled T_3_ as a proxy for T_3_ uptake, we used 25 ng of stable isotope-labeled rT_3_ as an internal standard to correct for extraction efficiency.

### Tissue Explant Culture and T_3_ Treatments

To further investigate if thyroid axis components in the *E. coqui* limb and tail are functional, we cultured *E. coqui* and *X. tropicalis* limb and tail explants (30, 31), treated them with T_3_, and assayed gene expression. We treated NF stage 52–54 (32) *X. tropicalis* tadpoles and TS stage 9 *E. coqui* embryos with 50 U/mL of penicillin-streptomycin added to aquarium or Petri dish solution for 24 h prior to dissection. Tadpoles and embryos were terminally anesthetized and dipped into 70% ethanol to sterilize the epidermis before dissection. Four *X. tropicalis* and two *E. coqui* individuals were pooled to make a single biological replicate of each species. Tissues were dissected into ice-cold 1:1.5-diluted Leibowitz-15 media (Gibco) containing 50 U/mL penicillin-streptomycin, 50 mg/mL gentamicin and 10 mM HEPES. Prior to T_3_ treatment tissues were cultured overnight in media supplemented with insulin (500 ug/mL) on a laboratory bench at room temperature (21°C) with gentle shaking (50 rpm). The next morning, stock T_3_ was diluted in 0.01 N NaOH and added to the media to a final concentration of 50 nM. Media and T_3_ were changed every 8–12 h. After treatment for 8 or 46 h, limb and tail explants were rinsed three times in phosphate-buffered saline (PBS) and homogenized in TriZol. RNA was isolated according to the manufacturer's protocol.

### Statistical Analysis

Statistical analyses of qPCR data were done with RStudio version 1.0.136 and visualized with ggplot2 (https://ggplot2.tidyverse.org/). Developmental timeline qPCR and iodothyronine content data followed a non-normal distribution as determined by Q-Q plots and the Shapiro-Wilk test; Levene's test determined that TH content data additionally had unequal variance. Log_10_-transformed data were not normally distributed. Therefore, a Kruskal-Wallis test was used to determine if there were significant differences among groups, and a *post-hoc* Dunn's test with the Benjamini and Hochberg (BH) correction was used to identify stages that differ from each other while adjusting for multiple comparisons. We performed a least squares regression on T_4_, T_3_, and rT_3_ data sets to investigate possible differences in iodothyronines kinetics during development. For the developmental timeline qPCR data, statistical tests were performed on data pooled from two independent experiments (see Supplementary Data for data from each experiment). For *in vivo* and *in vitro* T_3_ treatment experiments, Student's *t*-test was used to identify significant differences between T_3_-treated groups and controls.

## Results

### Predicted Proteins of Isolated *E. coqui* cDNAs Contain Conserved Domains

Most isolated cDNAs contained functional domains of orthologous proteins. The predicted *E. coqui* TRα and TRβ sequences cover amino acids 11–281 (65%), and amino acids 9–273 (69%) of the orthologous *X. tropicalis* proteins, respectively. Both predicted TR protein sequences contain the DNA-binding domain and most of the ligand-binding domain. Alignments show that the predicted protein sequence of the *E. coqui* TRα DNA-binding domain has 97% identity to the *X. tropicalis* DNA-binding domain, while the TRα ligand-binding domain shared between the predicted *E. coqui* and *X. tropicalis* sequences are 98% identical. The DNA-binding domain of the predicted *E. coqui* TRβ sequence is 100% identical to the DNA-binding domain in *X. tropicalis* TRβ, and the ligand-binding domain is 95% identical. The predicted partial *E. coqui* Dio2 sequence covers amino acids 2–254 (98%) of *X. tropicalis* Dio2 and the partial *E. coqui* Dio3 sequence covers amino acids 7–252 (90%) of *X. tropicalis* Dio3. Additionally, the predicted protein sequence of both *dio2* and *dio3* isolated cDNAs contain the selenocysteine site and the thioredoxin domain. Both thioredoxin domains share 86% identity with the orthologous *X. tropicalis* thioredoxin domain. The partial predicted amino acid sequence of *E. coqui Klf9* covers amino acids 194–264 (25%) of *X. tropicalis Klf9* and contains the three characteristic zinc-finger domains (100% identity) in the C-terminus of *X. tropicalis Klf9*. The isolated *E. coqui thibz* sequence covers amino acids 159–335 (53%) of *X. tropicalis* NFIL3-like (synonym for *thbzip*) and lacks the highly conserved basic leucine zipper domain. Even without the highly conserved basic leucine zipper domain, the predicted *E. coqui* protein sequence still clusters with other orthologous NFIL3-like proteins, rather than with other proteins with the basic leucine zipper domain (NFIL3 and CREB1) in maximum likelihood trees of these three orthologous vertebrate proteins (data not shown). Similarly, the other partial predicted *E. coqui* sequences cluster with other orthologous genes rather than with other closely related proteins containing similar domains (data not shown). We confirmed all isolated *E. coqui* cDNAs against the full-length transcript provided by investigators listed in the methods. Finally, we also performed BLASTx and BLASTn searches with the isolated *E. coqui* cDNA sequences. All cloned sequences have high similarity to predicted orthologous genes in frog species and other vertebrates (Supplementary Table 4).

### Changes in Whole Body Iodothyronine Content During Embryonic *E. coqui* Development

Using LC-MS/MS, we detected the iodothyronines T_4_, T_3_, and rT_3_ in unfertilized oocytes and at every stage of development (Figure 2). Thyroxine content (pg/mg body weight) was highest, followed by rT_3_ and then T_3_. We detected T_2_ only at TS stages 14 and 15, when hatching occurs, and at this point, T_2_ content was less than all other iodothyronine content and ranged between 0.04 and 0.78 pg/mg body weight (Supplementary Table 3). The three quantifiable iodothyronines were low and relatively constant up to TS stage 8, after which stage they showed statistically significant increases [T_3_: Kruskal-Wallis rank sum test, *X*^2^ = 43.2 (df = 15), *p* < 0.001; T_4_: Kruskal-Wallis rank sum test, *X*^2^ = 43.7 (df = 15), *p* < 0.001; rT_3_: Kruskal-Wallis rank sum test, *X*^2^ = 39.7 (df = 14), *p* < 0.001]. Whole body content of all three iodothyronines showed statistically significant increases between stages 8 and 13 (*post-hoc* Dunn's test; *p* = 0.048, 0.033, 0.035 for T_3_, T_4_, and rT_3_, respectively). The velocity of change was slower for rT_3_ and T_3_ compared with T_4_. Stage was a significant predictor for all three iodothyronines [T_3_: *F* = 70.8 (df = 45), *p* < 0.001; T_4_: *F* = 54.2 (df = 46) *p* < 0.001; rT_3_: *F* = 23.7 (df = 43), *p* < 0.001]. Although all iodothyronines are positively correlated with stage, the velocity of change was slower for rT_3_ and T_3_ (slope of least squares regression (LSR) line, b = 0.2964 and 0.1009, respectively) compared to T_4_ (LSR, b = 0.7984). Tissue content of all three iodothyronines was highest at TS 15. Note also that oocytes and early embryos (TS 0–5) of *E. coqui* have large yolk deposits, which may increase the S/N ratio and cause an underestimation of iodothyronine content in the embryo and yolk at these stages.

### Changes in Thyroid Hormone Receptor and Deiodinase mRNA Levels in the Embryonic Tail

Both *thra* and *thrb* mRNAs in the *E. coqui* tail showed statistically significant changes during development [Figure 3A; *thra*: Kruskal-Wallis rank sum test, *X*^2^ = 20.18 (df = 4), *p* < 0.001; *thrb*: Kruskal-Wallis rank sum test, *X*^2^ = 26.78 (df = 4), *p* < 0.001]. *Thyroid hormone receptor* α and *thrb* mRNA in the tail bud are approximately equal at TS stage 5 (Figure 3A). *Thyroid hormone receptor* α mRNA in the tail at hatching is between 2.1- and 4-fold higher than the early tail (TS stages 5 and 7, *post-hoc* Dunn's test, *p* = 0.002 and 0.03, respectively). *Thyroid hormone receptor* β mRNA follows a similar pattern—it increased 4-fold between the onset of tail resorption (TS 13) and hatching (TS 15)—although *thra* increased only 1.8-fold over the same interval (Figure 3A). *Thyroid hormone receptor* β mRNA at hatching (TS 15) is between 18- and 47-fold higher than in the early tail (TS stage 5 and TS stage 7, *post-hoc* Dunn's test, *p* < 0.001 and *p* = 0.002, respectively).

*Deiodinase type II* and *dio3* mRNAs significantly changed during tail development (Figure 3B; *dio2*: Kruskal-Wallis rank sum test, *X*^2^ = 17.37 (df = 4), *p* = 0.002; *dio3*: Kruskal-Wallis rank sum test, *X*^2^ = 26.11 (df = 4), *p* < 0.001). Patterns of deiodinase mRNA in the developing tail were essentially the opposite of those seen in the limb. *Deiodinase type II* mRNA was low throughout tail development and resorption but rose almost 10-fold as hatching neared (TS 15; Figure 3B). At hatching (TS 15), *dio2* mRNA was higher than at TS 5, 7 and 13 (*post-hoc* Dunn's test, *p* = 0.001, 0.029, and 0.031, respectively). *Deiodinase type III* mRNA increased 27-fold between TS 5 and 13 (*post-hoc* Dunn's test, *p* < 0.001) and then decreased steeply (11-fold) between the onset of tail resorption and hatching (*post-hoc* Dunn's test, *p* = 0.007). Repeated experiments demonstrate the similar patterns of *thra, thrb, dio2*, and *dio3* expression (Supplementary Figure 1).

### Changes in Thyroid Hormone Receptor and Deiodinase mRNA Levels in the Embryonic Hind Limb

**Figure 4:**
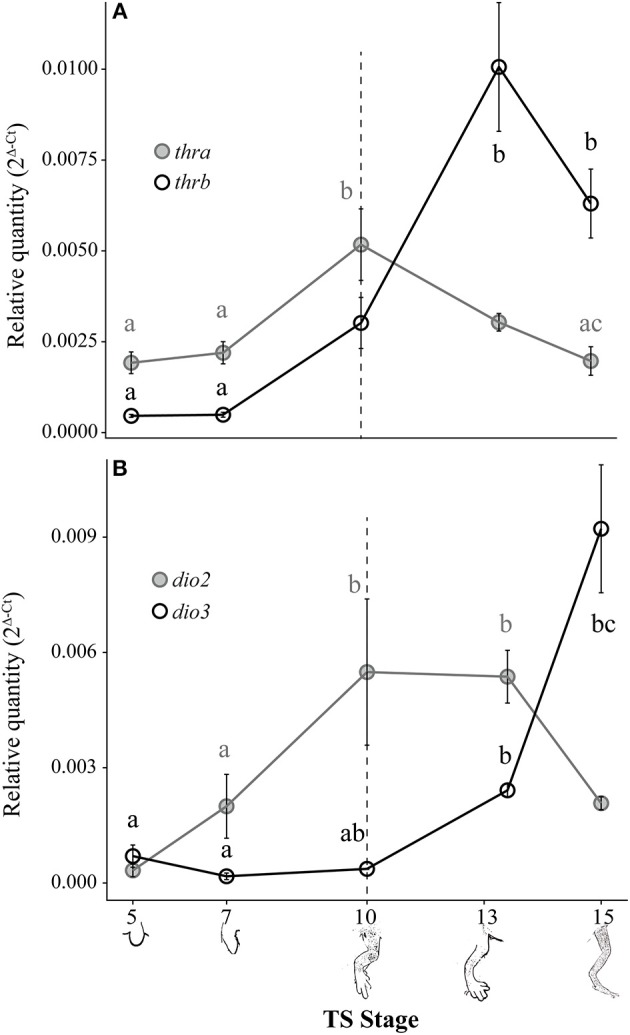
Relative *thra* and *thrb* mRNA levels **(A)** and *dio2* and *dio3* mRNA levels **(B)** in the pre-hatching hind limb of *E. coqui*. Dashed vertical line at TS stage 10 marks when thyroid follicles are first visible in histological sections. Drawings on the x-axis depict sequential formation of the limb. Each expression value is represented as a circle centered on the mean of 5–7 hind limb pairs ± SE. Lower-case letters in gray (*thra* and *dio2*) and black (*thrb* and *dio3*) indicate significant pairwise differences between groups (*post-hoc* Dunn's test, *p* < 0.05). See Supplementary Data for a complete list of pairwise comparisons.

Both *thra* and *thrb* mRNAs in the *E. coqui* hind limb showed statistically significant changes during development (Figure 4A; *thra*: Kruskal-Wallis rank sum test, *X*^2^ = 20.66 (df = 4), *p* < 0.001; *thrb*: Kruskal-Wallis rank sum test, *X*^2^ = 25.36 (df = 4), *p* < 0.001). The level of *thra* mRNA was greater than *thrb* mRNA in the limb bud until TS 10, when the *thra* mRNA level began to decrease and continued to decline through hatching (Figure 4A). The peak *thra* mRNA level at TS 10 coincides with the appearance of thyroid follicles (13); *thra* mRNA in the hind limb at this stage was significantly higher than in the limb bud at TS 5 (*post-hoc* Dunn's test, *p* = 0.001), in the limb paddle at TS stage 7 (*p* = 0.009) and in the fully formed froglet limb at TS 15 (*post-hoc* Dunn's test, *p* = 0.002). At hatching, *thra* mRNA level was lower than *thrb* mRNA levels. Between paddle (TS 7) and toepad formation (TS 13), *thrb* mRNA rose ~21-fold to a peak at TS 13. At TS 13, *thrb* expression was significantly higher than in the limb bud and paddle (Figure 4A; TS 5 and 7; *post-hoc* Dunn's test, *p* < 0.001 and p = 0.001, respectively). *Thyroid hormone receptor* β mRNA drops almost 1.5-fold between TS 13 and hatching.

*Deiodinase type II* and *dio3* mRNAs both showed statistically significant but contrasting patterns throughout limb development [Figure 4B; *dio2*: Kruskal-Wallis rank sum test, *X*^2^ = 18.65 (df = 4), *p* < 0.001; *dio3*: Kruskal-Wallis rank sum test, *X*^2^ = 25.76 (df = 4), *p* < 0.001]. *Deiodinase type II* mRNA increased 16-fold between limb bud (TS 5) and digit formation (TS 10) and remained at this level through subsequent limb growth (TS 13; *post-hoc* Dunn's test, *p* = 0.007 and *p* < 0.001, respectively). *Deiodinase type II* mRNA decreased 2.6-fold between TS 13 and hatching to the level originally present in the newly formed limb bud (e.g., TS 5). *Deiodinase type III* mRNA remained low throughout most of limb development, but it increased 25-fold between the initial formation of thyroid follicles (TS 10) and hatching (TS 15; *post-hoc* Dunn's test, *p* = 0.001). Repeated experiments show the same general contrasting mRNA expression patterns for *dio2, dio3, thra*, and *thrb* (Supplementary Figure 2).

### Exogenous T_3_ Induced Gene Expression Responses in the TS 9 *E. coqui* Tail, but Not the Limb

**Figure 5:**
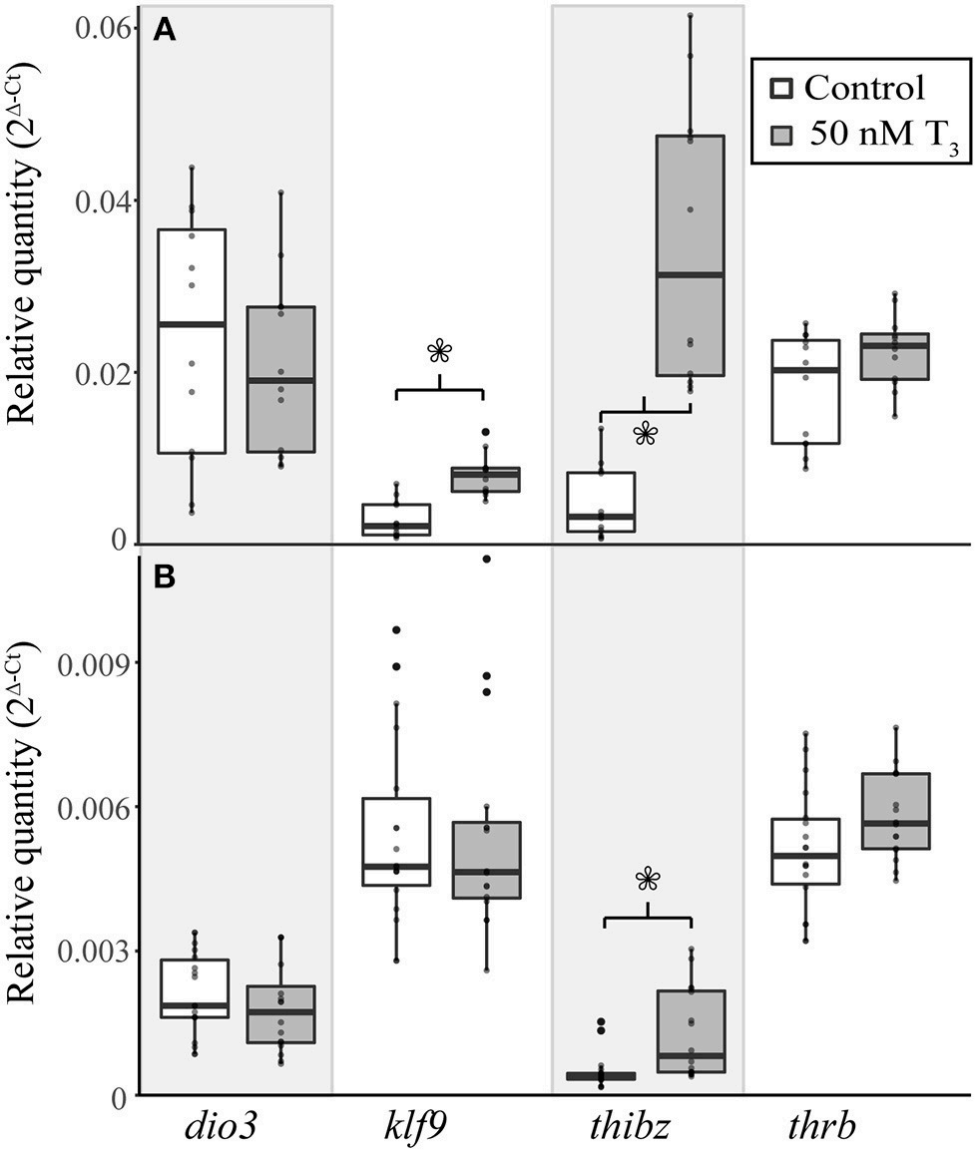
Exogenous treatment with 50 nM T_3_ for 8 h induces gene expression in the tail of *E. coqui* embryos at TS stage 9 **(A)**, but not in limbs at the same stage **(B)**. Boxes and whiskers depict the median and range of 12–16 individuals from two independent experiments. Asterisks indicate a significant change in expression (Student's *t*-test, *p* < 0.05).

To determine if *E. coqui* tissues are capable of mounting a gene regulation response to exogenous T_3_, we performed *in vivo* T_3_ treatments (Figure 5A). Immersion of TS 9 *E. coqui* embryos in 50 nM T_3_ for 8 h caused a significant induction of *klf9* (Student's *t*-test, *t* = 5.61 (df = 21.74), *p* < 0.001) and *thibz* (Student's *t*-test, *t* = 6.20 (df = 12.42), *p* < 0.001) in the tail. Immersion in 50 nM T_3_ for 46 h additionally significantly induced *thrb* mRNA (Supplementary Figure 4). In contrast, the identical treatment significantly increased only *thibz* expression (Figure 5B; Student's *t*-test, *t* = 3.11 (df = 18.92), *p* = 0.006) in the limb.

### *E. coqui* Embryos Took up Significantly Less T_3_ From the Environment Than Did *X. tropicalis* Tadpoles

Because previous studies suggested that *E. coqui* limbs are insensitive to TH, and because we observed a weak TH response in our *in vivo* experiments, we wanted to confirm that immersion in T_3_ increased tissue content of T_3_. We quantified stable isotope-labeled T_3_ tissue content after immersing *X. tropicalis* tadpoles or *E. coqui* embryos in stable isotope-labeled T_3_ solution under three conditions. We chose 50 nM T_3_ and 46 h treatment to match the *E. coqui in vivo* T_3_ response experiments. We also chose two conditions that represent relevant time points from previous studies of larval *Xenopus* species: (1) treatment with 1 nM T_3_ for 8 h is sufficient for *X. tropicalis*' whole body T_3_ content to surpass the T_3_ concentration in the surrounding media (33), and (2) treatment with 1 nM T_3_ for 24 h is sufficient to induce gene expression responses in *X. tropicalis* (31, 34). After immersing *E. coqui* in 1 nM labeled T_3_ for 8 and 24 h, we detected endogenous T_3_ but not labeled T_3_. However, we detected labeled T_3_ in *X. tropicalis* tissue at both 8 and 24 h (Table 2). We detected stable isotope-labeled T_3_ in both *E. coqui* and *X. tropicalis* tissue following 46-h treatment with 50 nM T_3_. Total content of labeled T_3_ in *X. tropicalis* tissue was ~63 times that found in *E. coqui* tissues [Table 2, Student's *t*-test, *t* = −3.20 (df = 2.00), *p* = 0.085]. Additionally, *X. tropicalis* has ~ 875 times more stable isotope-labeled T_3_ than endogenous T_3_ content. In contrast, stable isotope-labeled T_3_ in *E. coqui* is approximately equal to endogenous T_3_ content.

**Table 2:** Nieuwkoop and Faber stage 51–55 *Xenopus tropicalis* tadpoles have more labeled T_3_ tissue content than do TS stage 9 *E. coqui* embryos after immersion in labeled T_3_ for 8, 24, or 46 h.

			**Labeled T_**3**_**	**T_**3**_**
**Species**	**Labeled T_**3**_ concentration (nM)**	**Timepoint (h)**	**pg/mg**	**pg/mg**
*X. tropicalis*	1	8	1.079 ± 0.19	0.095 ± 0.02
*E. coqui*	1	8	0.000 ± 0.00	0.236 ± 0.01
*X. tropicalis*	1	24	1.371 ± 0.08	0.018 ± 0.01
*E. coqui*	1	24	0.000 ± 0.00	0.245 ± 0.04
*X. tropicalis*	50	46	30.436 ± 9.37	0.035 ± 0.01
*E. coqui*	50	46	0.483 ± 0.27	0.447 ± 0.20

*Each value represents the mean of 3–6 individuals ± standard error*.

### Exogenous T_3_ Strongly Induced T_3_ Response Genes in TS Stage 9 *E. coqui* Limb Explants

**Figure 6:**
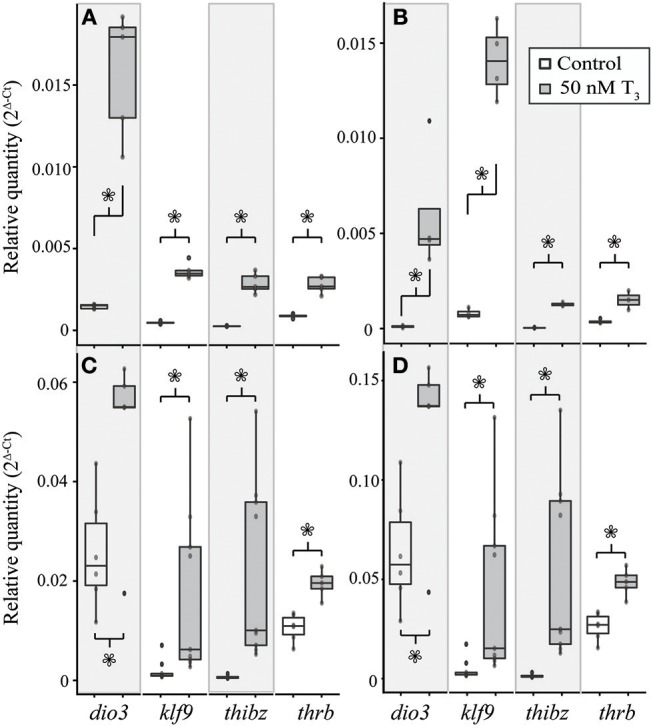
Treatment with 50 nM exogenous T_3_ for 8 h induces *deiodinase type III* (*dio3*), *krüppel-like factor 9* (*klf9*), *thyroid hormone induced bZip protein* (*thibz*), and *thrb* expression in NF stage 52–54 *X. tropicalis* tail **(A)** and limb **(B)** explants and in TS stage 9 *E. coqui* tail **(C)** and limb explants **(D)**. Asterisks indicate a significant increase in mRNA levels (Student's *t*-test, *p* < 0.05).

Treatment with 50 nM T_3_ for 8 h significantly increased *dio3* [Student's *t*-test, *t* = 8.40 (df = 4.00), *p* = 0.001), *klf9* (Student's *t*-test, *t* = 14.41 (df = 4.18), *p* < 0.001], *thibz* [Student's *t*-test, *t* = 9.64 (df = 4.01), *p* < 0.001], and *thrb* [Student's *t*-test, *t* = 8.26 (df = 4.39), *p* < 0.001] mRNAs in explants of *X. tropicalis* tail (Figure 6A). The same treatment caused a significant increase in *dio3* [Student's *t*-test, *t* = 3.49 (df = 3.00), *p* = 0.040], *klf9* [Student's *t*-test, *t* = 13.66 (df = 3.08), *p* < 0.001], and *thibz* [Student's *t*-test, *t* = 21.50 (df = 2.07), *p* = 0.002] mRNAs in *X. tropicalis* limb explants (Figure 6B). Similarly, exogenous T_3_ increased *dio3* [Student's *t*-test, *t* = 2.56 (df = 6.47), *p* = 0.040], *klf9* [Student's *t*-test, *t* = 2.67 (df = 8.22), *p* = 0.028], *thibz* [Student's *t*-test, *t* = 3.54 (df = 8.00), *p* = 0.008], and *thrb* [Student's *t*-test, *t* = 5.38 (df = 8.62), *p* < 0.001] mRNAs in explants of *E. coqui* tail (Figure 6C). *Deiodinase type III* [Student's *t*-test, *t* = 2.61 (df = 9.80), *p* = 0.027], *klf9* [Student's *t*-test, *t* = 6.11 (df = 8.05), *p* < 0.001], *thibz* [Student's *t*-test, *t* = 6.49 (df = 8.00), *p* < 0.001], and *thrb* [Student's *t*-test, *t* = 7.70 (df = 8.80), p < 0.001] increased after the same treatment in *E. coqui* limb explants (Figure 6D).

In both species, the magnitude of increase for all genes was greater in the limb than in the tail (Table 3). The same trends were observed after treating tissue explants with 50 nM T_3_ for 46 h (Supplementary Figure 5). In tail explants, T_3_ induced fold changes of a similar order of magnitude for *thibz* (between 42- and 44-fold) and *thrb* (between 1.8- and 3.8-fold), but not for *dio3* and *klf9*; in *E. coqui, dio3*, and *klf9* mRNAs increased 2- and 9.9-fold, respectively, while *dio3* and *klf9* mRNAs increased 11.9- and 12.5-fold in *X. tropicalis*. In limb explants, *dio3* and *thibz* mRNA differed by an order of magnitude between species. *Deiodinase type III* mRNA increased 58.8-fold in *X. tropicalis* limb explants, while *dio3* mRNA increased 3.7-fold in *E. coqui* limb tissue. *Thyroid hormone induced bZip protein* mRNA increased only 37-fold in *X. tropicalis* limb explants, while *dio3* mRNA increased 180-fold in *E. coqui* limb explants.

**Table 3:** Induction of *deiodinase type III* (*dio3*), *krüppel-like factor 9* (*klf9*), *thyroid hormone induced bZip protein* (*thibz*), and *thyroid hormone receptor* β (*thrb*) in tail and limb explants of NF stages 52–54 *Xenopus tropicalis* and TS stage 9 *Eleutherodactylus coqui* after treatment with 50 nM T_3_ for 8 h.

		**Average fold increase**	
**Species**	**Gene**	**Tail**	**Limb**
*X. tropicalis*	*dio3*	11.9	58.8
	*klf9*	12.5	17.8
	*thibz*	43.8	37.1
	*thrb*	3.8	4.0
*E. coqui*	*dio3*	2.0	3.7
	*klf9*	9.9	21.3
	*thibz*	42.0	180.0
	*thrb*	1.8	3.0

*Values represent the average fold increase above control (vehicle-treated) levels*.

## Discussion

In this study we show that the core TH signaling components are evolutionarily conserved in *Eleutherodactylus coqui* limb and tail tissue. We also show that developmental patterns of *thra, thrb, dio2*, and *dio3* mRNAs, and whole-body TH content in *E. coqui* closely match those reported during metamorphosis of *Xenopus* species. We also find maternal T_4_, T_3_, and rT_3_ in unfertilized eggs and early embryos of *E. coqui*, which may mediate TR signaling prior to embryonic thyroid gland formation. This is the first published report of TH metabolites and maternally derived TH in a direct-developing frog. Additionally, we demonstrate that *E. coqui* tissues show robust gene expression responses to exogenous T_3_ similar to those seen in metamorphosing species. *Eleutherodactylus coqui* embryos take up much less T_3_ from the environment compared with *X. tropicalis*. This difference likely explains the relatively weak and variable gene expression responses seen *in vivo* in *E. coqui*, and was likely a significant confounding factor for previously published results.

### Developmental Profiles of Whole Body Iodothyronine Content

Temporal dynamics of whole-body iodothyronine content in direct-developing *E. coqui* mirror those described for indirect-developing frogs, which retain the ancestral biphasic life history: *Scaphiopus hammondii* (28), *Rana catesbeiana* (35), *Bufo marinus* (36), *Bufo japonicus* (37), and *Xenopus laevis* (33). Anuran metamorphosis comprises three successive stages: premetamorphosis, when little to no TH is present; prometamorphosis, when TH concentrations slowly rise; and a rapid metamorphic climax characterized by a peak in TH concentrations. The temporal profile of TH content in embryonic *E. coqui* similarly defines three successive periods: (1) Low TH content characterizes the first half of development, prior to thyroid follicle formation (TS 1–8). (2) After thyroid follicles appear, TH content gradually rises until tail resorption began (TS 9–12). (3) TH content dramatically increases, with a peak in TH at or just prior to hatching (TS 13–15). In addition to amphibians, many other vertebrates experience peak concentrations of TH at life history transitions—at hatching in precocial birds (38), at the larval-to-juvenile transition in several fish species (39–41), at ~14 days post-partum in rats and mice (42, 43), and at birth in humans (44).

Thyroid hormones are present throughout early embryogenesis and the subsequent period of pre-hatching development in *E. coqui* (TS 1–9), beginning up to eight days before thyroid follicles can be detected histologically (13). These hormones are almost certainly maternal in origin. Similarly, T_4_ and T_3_ have been detected in yolk and gastrulating embryos of four other anuran species—*Bufo marinus* (36), *Rana catesbeiana* (35), *Bombina orientalis* (45), and *Xenopus laevis* (16). Early *Xenopus tropicalis* embryos express key TH signaling components (46). Indeed, TH signaling is also functional in the *Xenopus* tadpole central nervous system (CNS) before thyroid gland formation (16, 18). Maternally derived TH has a conserved role in vertebrate CNS development (47) and embryogenesis (17, 48, 49). Therefore, it seems likely that direct-developing frogs require maternal TH for normal neural development, as do most vertebrate species, although we do not evaluate that hypothesis here.

Maternal TH may regulate limb development occurring before the differentiation of the embryonic thyroid gland in direct-developing frogs. In metamorphosing anurans, TH signaling is required for terminal limb differentiation (22), but the initial stages of limb development are TH-independent. For example, tadpoles immersed in methimazole, a TH-synthesis inhibitor, develop a long limb-bud-like structure (24), and thyroidectomized tadpoles develop calcification centers in the hind limb (50, 51). In *E. coqui*, the limb bud proliferates and digits develop prior to the appearance of embryonic thyroid follicles (TS stages 9–10) [Figure 3; (8, 13)]. Two hypotheses could account for this observation: (1) *E. coqui* relies on maternal TH, rather than embryonically produced TH, to regulate early stages of digit patterning and growth (TS 6–9); or (2) paddle and digit formation in *E. coqui* proceed independently of TH. Our data show that requisite components of TH signaling are present at this time. Future investigation should evaluate the functional role of TH during this critical developmental period. A switch from embryonic to maternally synthesized TH for the regulation of early limb development, if it occurred, could explain the heterochronic shift in limb development and would represent an evolutionary novelty in direct-developing species.

### Thyroid Hormone Receptor α, thrb, Dio2, and Dio3 mRNA Expression Patterns During Development and T3 Response in the Embryonic Tail

Tail resorption in *Xenopus tropicalis* occurs late in metamorphosis and is mediated by TRβ (52). Because tail resorption in *E. coqui* occurs late in embryogenesis and requires T_3_ (8), we expected that *thra, thrb, dio2*, and *dio3* mRNA dynamics in the *E. coqui* tail would mirror those described in *Xenopus*. Our results support this hypothesis: in the *E. coqui* tail, a rise in *thrb* expression coincides with the rise in embryonic TH content, consistent with a role for *thrb* in mediating tail resorption.

*Deiodinase type II* and *dio3* mRNA expression patterns in the developing *E. coqui* tail are also similar to those described in indirect-developing species in which these deiodinase enzymes are critical for coordinating metamorphosis (20). Elevated *dio3* expression protects the tail from an early apoptotic response to T_3_ until metamorphic climax in *Xenopus* (26); *E. coqui* tail resorption also begins at TS 13, when *dio3* expression significantly decreases. Although they serve different functions, the tail serves a critical role in both species: the larval *Xenopus* tail is a critical locomotor organ, whereas the embryonic *E. coqui* tail functions in respiration. In both species, maintenance of the tail is accomplished in part by *dio3* inactivation of T_4_ and T_3_.

Given the conservation of mRNA dynamics in the *E. coqui* tail, we wanted to determine whether the tissue could respond to exogenous T_3_. In *Xenopus* species, treatment with exogenous T_3_ induces transcription of direct T_3_ response genes *dio3, klf9, thibz*, and *thrb* (19, 53–56). Exogenous T_3_ induces significant increases in the mRNA of three of these T_3_ response genes, *klf9, thibz*, and *thrb*, supporting the hypothesis that TH signaling components are conserved and mediate tail resorption in *E. coqui*.

### Thyroid Hormone Receptor α, thrb, Dio2, and Dio3 mRNA Expression Patterns During Development and T_3_ Response in the Embryonic Hind Limb

*Thyroid hormone receptor* α*, thrb, dio2*, and *dio3* mRNA expression patterns parallel those described in *Xenopus* species in the period leading up to and during metamorphosis (33, 57). In indirect-developing frogs, TRα has a critical role in controlling post-embryonic developmental timing (58–60) and in promoting proliferation in the hind limb during metamorphosis (61–63). Constitutive *thra* expression supports a proliferative and competence-establishing role for TRα in *E. coqui*. In the *E. coqui* limb, a rise in *thrb* expression coincides with the rise in embryonic TH content, consistent with TRβ autoinduction and tissue sensitization to TH described in *Xenopus* (64).

The tissue-specific patterns of *dio2* and *dio3* underlie the differential sensitivity of limb and tail tissue in metamorphosing frogs. *Deiodinase type II* expression is constitutive in the developing limb of *Xenopus laevis*, causing the limb to be sensitive to small amounts of T_3_ produced during premetamorphosis (23). Similarly, elevated *dio2* expression in *E. coqui* limbs throughout most of limb development, including several days prior to formation of the embryonic thyroid gland, supports a role for TH-mediated limb development and growth.

In indirect-developing species, including *Xenopus* and spadefoot toads (*Scaphiopus*), concentrations between 1 and 10 nM T_3_ are sufficient to promote precocious metamorphosis, tail resorption, and gene expression responses in limbs and tail (31, 65, 66). However, previous studies report that the *E. coqui* limb has no morphological response to high doses of exogenous T_3_ (11). Our study is the first to characterize mRNA expression changes in a direct-developing frog species in response to exogenous T_3_. Treatment of *E. coqui* embryos with exogenous T_3_ prior to formation of the thyroid follicles increases expression of four direct T_3_ response genes in the tail, consistent with studies in *Xenopus* species (19, 53–55). However, limbs of the same embryos do not respond to T_3_, despite the high dose administered (50 nM T_3_). The lack of response previously observed in direct-developing species may be confounded by an inability of T_3_ to reach the limb tissue. We observe a weak induction of T_3_ response genes in TS stage 7 limbs, a full two days before *E. coqui* begins to produce TH (Supplementary Figure 3). It is possible that this response occurs because the adult epidermis is not yet fully formed and T_3_ is better able to penetrate into the tissue, or because there is less endogenous T_3_ present at TS 7 than at TS 9. In either case, the ability to respond to T_3_ prior to thyroid gland formation is similar to biphasic species; tadpoles are also TH competent as soon as they hatch. Finally, the similar robust gene regulation response induced in *E. coqui* and *X. tropicalis* limb explants suggests that the limb tissue itself is similarly competent in both species. Overall, these data support the hypothesis that TH plays a role in *E. coqui* limb development and may do so prior to formation of the embryonic thyroid gland.

Here we support previous claims that later stages of limb development in *E. coqui* are TH-dependent but we additionally show that TH-signaling components are present during earlier stages, and that *E. coqui* limb tissue is sensitive to T_3_. *Eleutherodactylus coqui* eggs are provisioned with maternally derived TH, which may mediate organogenesis before differentiation and activity of the embryo's own thyroid gland. Altogether, our data suggest that the TH-mediated molecular module active during post-hatching metamorphosis in indirect-developing frogs has been shifted prior to hatching in direct-developing species.

## Ethics Statement

This study was carried out in accordance with the recommendations of the Harvard Faculty of Arts and Sciences Institutional Animal Care and Use Committee. The protocol was approved by the Harvard Faculty of Arts and Sciences Institutional Animal Care and Use Committee.

## Author Contributions

ML designed experiments and performed experiments, interpreted data, and wrote the manuscript. RD and JH contributed to experimental design, edited the manuscript, and discussed data interpretation.

### Conflict of Interest Statement

The authors declare that the research was conducted in the absence of any commercial or financial relationships that could be construed as a potential conflict of interest.
