# Supplementary material for: Evolutionary Conservation of Thyroid Hormone Receptor and Deiodinase Expression Dynamics in ovo in a Direct-Developing Frog, Eleutherodactylus coqui
Source: Front Endocrinol (Lausanne). 2019 May 24;10:307. doi: 10.3389/fendo.2019.00307 (PMC6542950; doi:10.3389/fendo.2019.00307)
Supplement: Supplementary file 2 [file Data_Sheet_1.pdf]

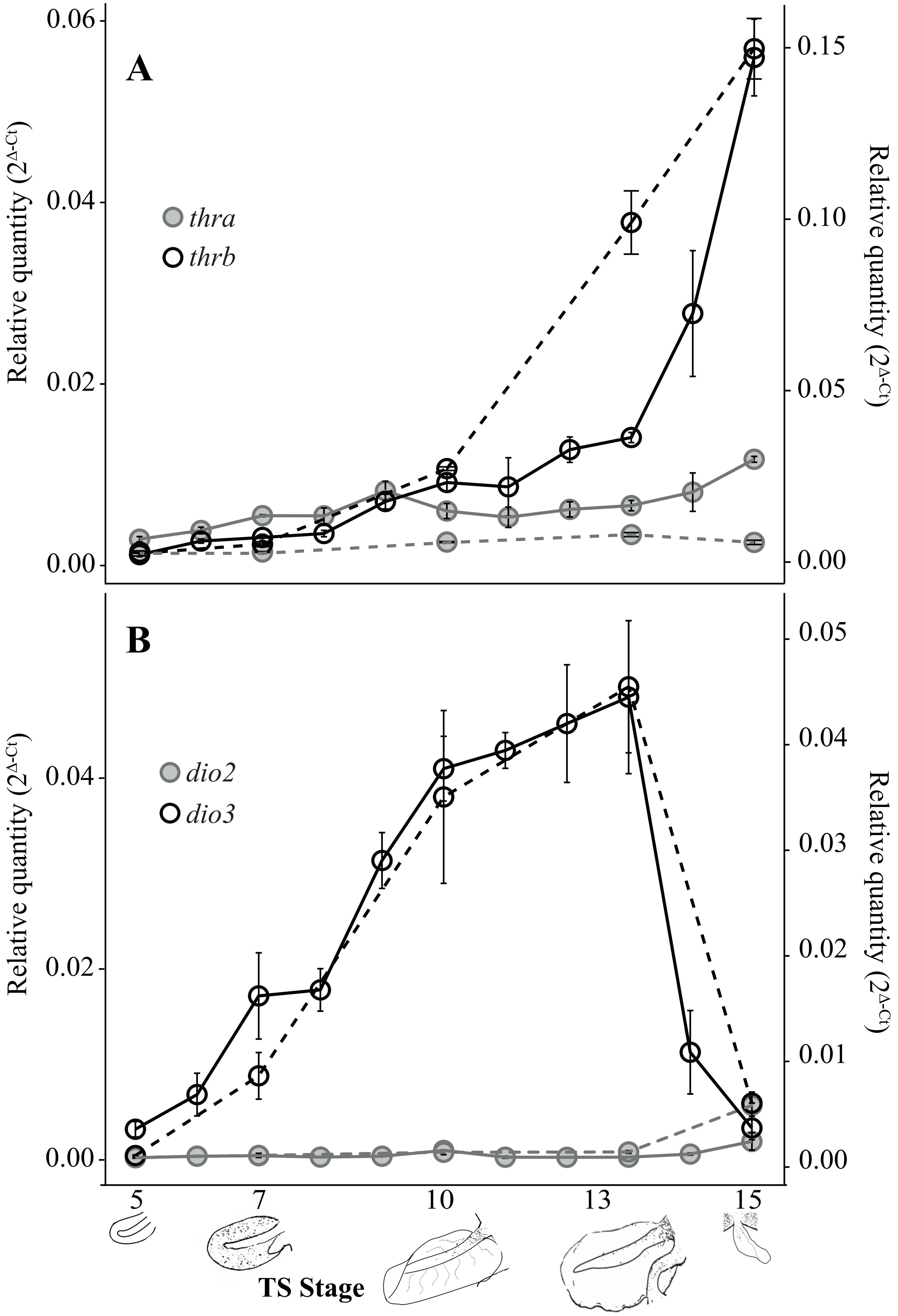

Supplementary Figure 1. Relative *thra* and *thrb* mRNA levels (A) and *dio2* and *dio3* mRNA levels (B) in the pre-hatching tail of *E. coqui*. Dashed lines connecting mean expression values indicate a second experimental replicate corresponding to the values on the y-axis on the right side of each plot. Drawings on the x-axis depict tail growth and resorption before hatching. Each expression value is represented as a circle centered on the mean of 3–4 individuals ± SE. See supplementary data for a complete list of significant pairwise comparisons for each independent experiment.

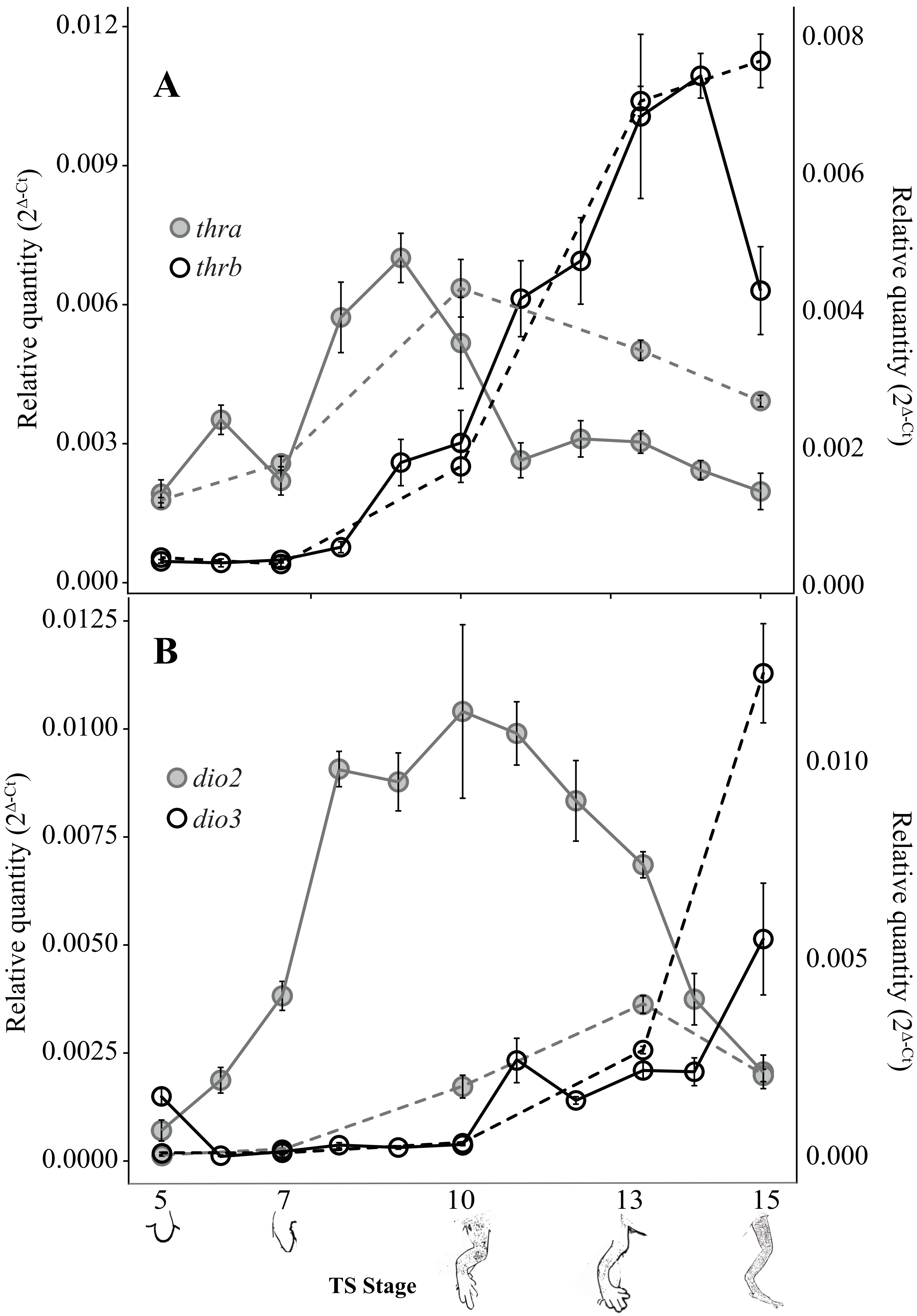

Supplementary Figure 2. Relative *thra* and *thrb* mRNA levels (A) and *dio2* and *dio3* mRNA levels (B) in the pre-hatching hind limb of *E. coqui*. Dashed lines connecting mean expression values indicate a second experimental replicate corresponding to the values on the y-axis on the right side of each plot. Drawings on the x-axis depict sequential formation of the limb. Each expression value is represented as a circle centered on the mean of 3–4 hind limb pairs ± SE. See supplementary data for a complete list of significant pairwise comparisons for each independent experiment.

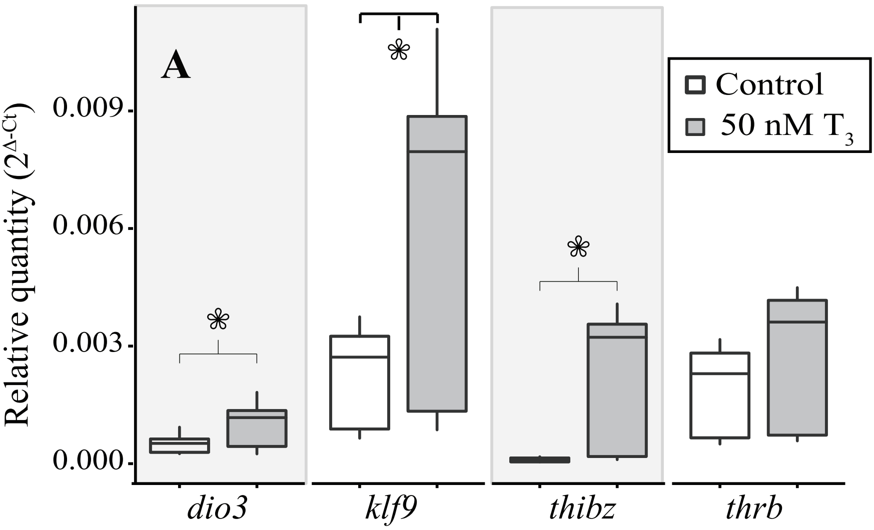

Supplementary Figure 3. Treatment with 50 nM exogenous T_3 for 24 hours induces *klf9*, *thibz*, and *thrb* expression in TS stage 7 *E. coqui* limb (A). Boxes and whiskers depict the median and range of 9–10 individuals from two independent experiments. Asterisks indicate a significant increase in mRNA levels (Student's t-test, $p < 0.05$).

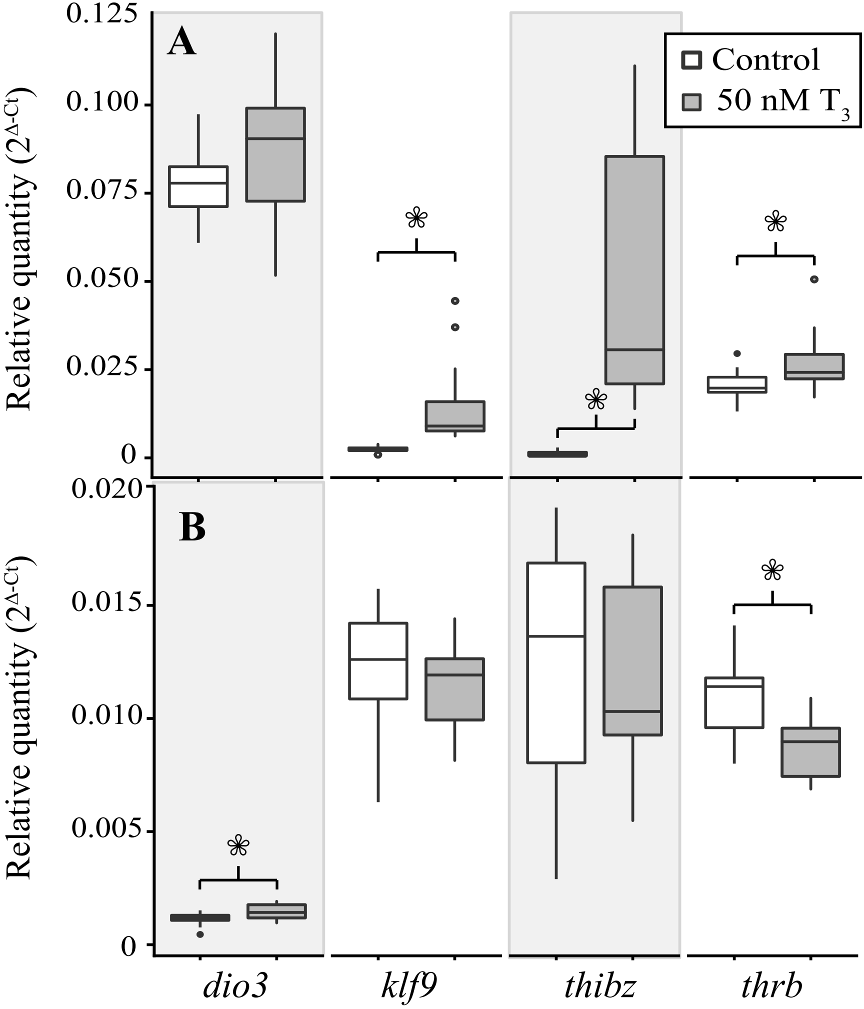

Supplementary Figure 4. Exogenous treatment with 50 nM T_3 for 46 hours induces an expression response in the tail of *E. coqui* embryos at TS stage 9 (A), but not in limbs at the same stage (B). Boxes and whiskers depict the median and range of 6–9 individuals from two independent experiments. Asterisks indicate a significant change in expression (Student's t-test, $p < 0.05$).

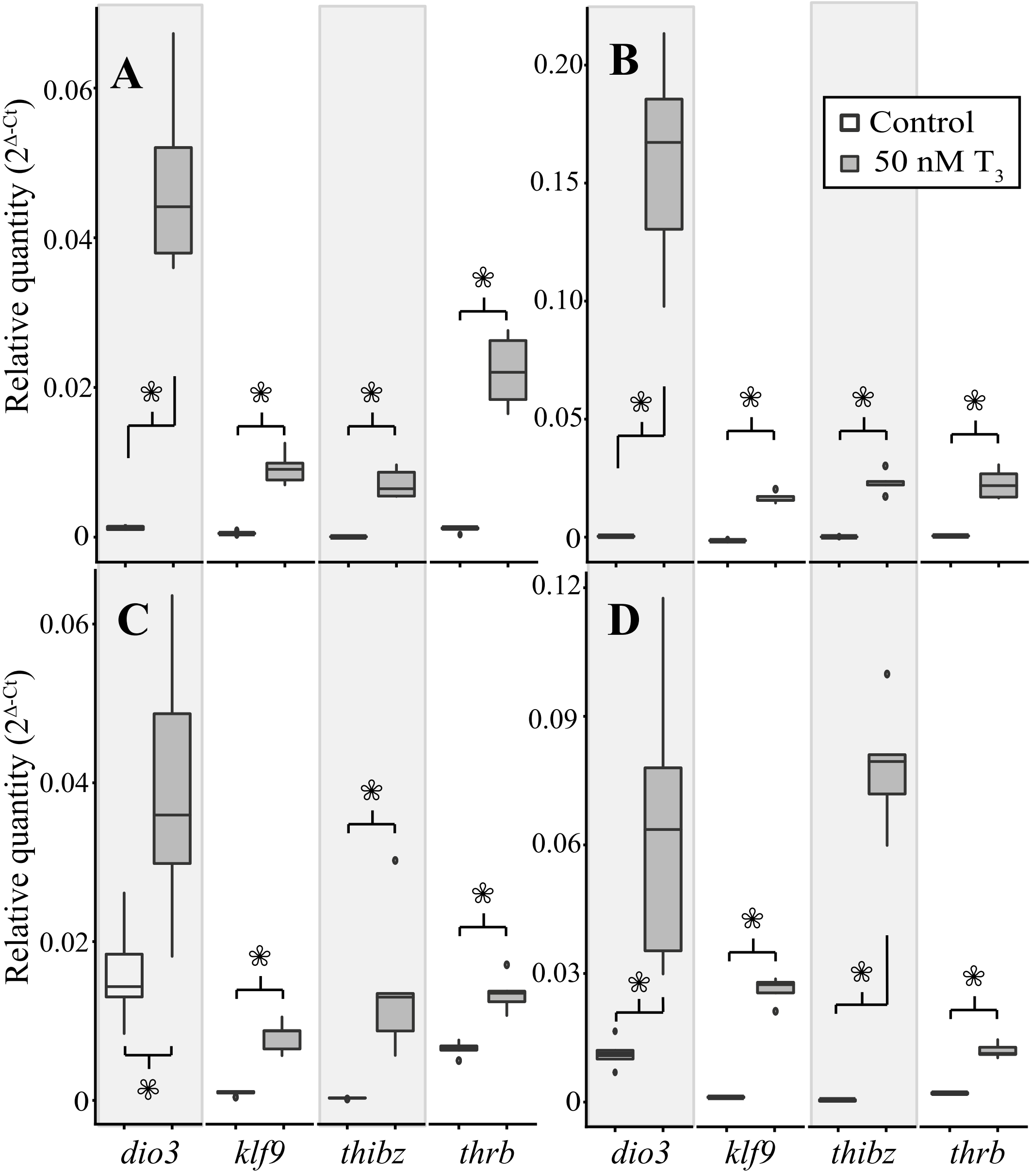

Supplementary Figure 5. Treatment with 50 nM exogenous T_3 for 46 hours induces *dio3*, *klf9*, *thibz* and *thrb* expression in NF stage 52–54 *X. tropicalis* tail (A) and limb (B) explants and in TS stage 9 *E. coqui* tail (C) and limb explants (D). Boxes and whiskers depict the median and range of 3–6 individuals. Asterisks indicate a significant increase in mRNA levels (Student's t-test, $p < 0.05$).
